# Flow damping due to stochastization of the magnetic field

**DOI:** 10.1038/ncomms6816

**Published:** 2015-01-08

**Authors:** K. Ida, M. Yoshinuma, H. Tsuchiya, T. Kobayashi, C. Suzuki, M. Yokoyama, A. Shimizu, K. Nagaoka, S. Inagaki, K. Itoh, the LHD Experiment Group, T. Akiyama, M. Emoto, T. Evans, A. Dinklage, X. Du, K. Fujii, M. Goto, T. Goto, M. Hasuo, C. Hidalgo, K. Ichiguchi, A. Ishizawa, M. Jakubowski, K. Kamiya, H. Kasahara, G. Kawamura, D. Kato, M. Kobayashi, S. Morita, K. Mukai, I. Murakami, S. Murakami, Y. Narushima, M. Nunami, S. Ohdach, N. Ohno, M. Osakabe, N. Pablant, S. Sakakibara, T. Seki, T. Shimozuma, M. Shoji, S. Sudo, K. Tanaka, T. Tokuzawa, Y. Todo, H. Wang, H. Yamada, Y. Takeiri, T. Mutoh, S. Imagawa, T. Mito, Y. Nagayama, K. Y. Watanabe, N. Ashikawa, H. Chikaraishi, A. Ejiri, M. Furukawa, T. Fujita, S. Hamaguchi, H. Igami, M. Isobe, S. Masuzaki, T. Morisaki, G. Motojima, K. Nagasaki, H. Nakano, Y. Oya, Y. Suzuki, R. Sakamoto, M. Sakamoto, A. Sanpei, H. Takahashi, M. Tokitani, Y. Ueda, Y. Yoshimura, S. Yamamoto, K. Nishimura, H. Sugama, T. Yamamoto, H. Idei, A. Isayama, S. Kitajima, S. Masamune, K. Shinohara, P. S. Bawankar, E. Bernard, M. von Berkel, H. Funaba, X. L. Huang, T. Ii, T. Ido, K. Ikeda, S. Kamio, R. Kumazawa, C. Moon, S. Muto, J. Miyazawa, T. Ming, Y. Nakamura, S. Nishimura, K. Ogawa, T. Ozaki, T. Oishi, M. Ohno, S. Pandya, R. Seki, R. Sano, K. Saito, H. Sakaue, Y. Takemura, K. Tsumori, N. Tamura, H. Tanaka, K. Toi, B. Wieland, I. Yamada, R. Yasuhara, H. Zhang, O. Kaneko, A. Komori

**Affiliations:** 1 National Institute for Fusion Science, Toki, Gifu 509-5292, Japan; 2 Research Institute for Applied Mechanics, Kyushu University, Kasuga 816-8580, Japan; 3 General Atomics, San Diego, California, USA.; 4 Max-Planck-Institut für Plasmaphysik, Wendelsteinstr. 1, 17489 Greifswald, Germany.; 5 The Graduate University for Advanced Studies, 322-6 Oroshi, Toki, Gifu 509-5292, Japan.; 6 Department of Mechanical Engineering and Science,Graduate School of Engineering, Kyoto University, Kyoto 606-8501, Japan.; 7 Laboratorio Nacional de Fusion, Asociacion EURATOM-CIEMAT, 28040 Madrid, Spain.; 8 Japan Atomic Energy Agency, Naka, Ibaraki 311-0193, Japan.; 9 Department of Nuclear Engineering, Kyoto University, Kyoto 606-8501, Japan.; 10 Department of Energy Engineering and Science, Nagoya University, Furo-cho, Chikusa, Nagoya 464-8603, Japan.; 11 Princeton Plasma Physics Laboratory, PO Box 45, Princeton, New Jersey 08543-0451, USA.; 12 Graduate School of Frontier Sciences, The University of Tokyo, 5-1-5 Kashiwanoha, Kashiwa-shi, Chiba-ken 277-8561, Japan.; 13 Department of Applied Mathematics and Physics Faculty of Engineering, Tottori University, 4-101 Koyama-Minami, Tottori 680-8552, Japan; 14 Institute of Advanced Energy, Kyoto University, Kyoto 611-0011, Japan; 15 Radioscience Research Laboratory, Faculty of Science,Shizuoka University, 836 Oya, Suruga-ku, Shizuoka 422-8529, Japan.; 16 Plasma Research Center, University of Tsukuba, 1-1-1 Tennodai, Tsukuba, Ibaraki 305-8577, Japan.; 17 Kyoto Institute of Technology, Matsugasaki, Sakyo-ku, Kyoto 606-8585, Japan.; 18 Graduate School of Engineering, Osaka University, 1-1 Yamadaoka, Suita, Osaka 565-0871, Japan.; 19 Research Institute for Applied Mechanics, Kyushu University, 6-1 Kasuga-kouen, Kasuga, Fukuoka 816-8580, Japan.; 20 Department of Quantum Science and Energy Engineering, Tohoku University, Sendai 980-8579, Japan.

## Abstract

The driving and damping mechanism of plasma flow is an important issue because flow shear has a significant impact on turbulence in a plasma, which determines the transport in the magnetized plasma. Here we report clear evidence of the flow damping due to stochastization of the magnetic field. Abrupt damping of the toroidal flow associated with a transition from a nested magnetic flux surface to a stochastic magnetic field is observed when the magnetic shear at the rational surface decreases to 0.5 in the large helical device. This flow damping and resulting profile flattening are much stronger than expected from the Rechester–Rosenbluth model. The toroidal flow shear shows a linear decay, while the ion temperature gradient shows an exponential decay. This observation suggests that the flow damping is due to the change in the non-diffusive term of momentum transport.

## Abstract (web summary)

Understanding the transport of ions, electrons and heat in magnetized plasmas is important to the development of fusion power as well as our understanding of the behaviour of astrophysical objects. Ida *et al.* find that stochastization of magnetic field lines in a plasma damps plasma flow more strongly than expected.

## Graphical abstract

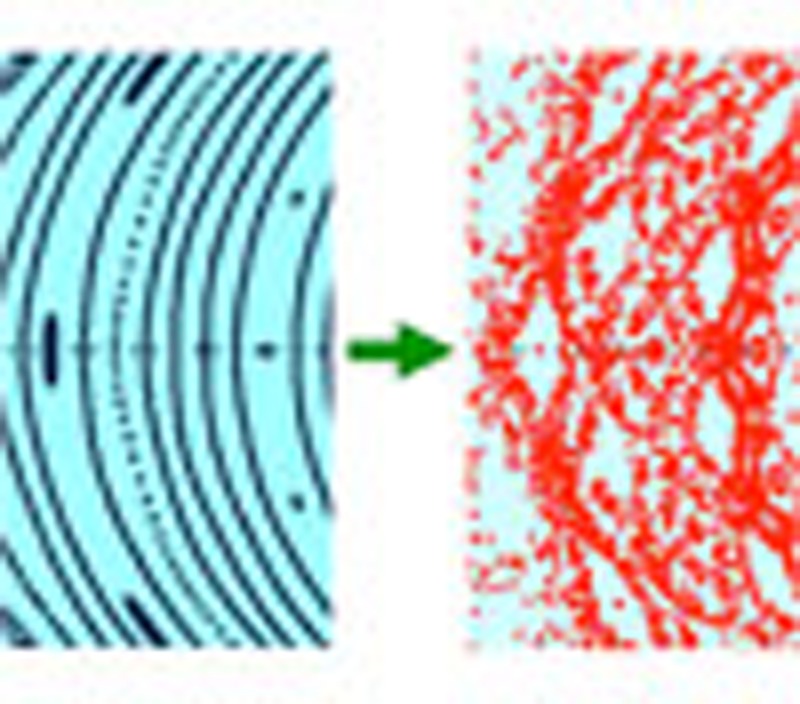

Stochastization of the magnetic flux surface is expected to be induced when the magnetic islands are overlapped and their width exceeds a threshold in toroidal plasmas. Stochastization of magnetic surfaces has been considered to be important because this mechanism, caused by perturbation fields, has a strong impact on transport and MHD events, such as a major disruption or an edge localized mode crash. The role of stochasticity in electron and ion heat transport has been studied in reverse field pinch (RFP) plasmas (in a reversed field experiment (RFX)^1^,^2^ and in the Madison Symmetric Torus (MST)^3^,^4^,^5^), where magnetic islands overlap and field lines are stochastic. In general, good agreement between the electron thermal diffusivity estimated from power balance and the analytic predictions of the Rechester–Rosenbluth model^6^ has been reported. However, the role of stochastization of the magnetic field in plasma flow has not been discussed before, in spite of the importance of flow shear in the turbulence in plasma, which determines the transport in toroidal magnetized plasmas, such as in tokamak, helical and RFP plasmas.

Here we demonstrate that stochastization of the magnetic field occurs when the magnetic shear at the rational surface decreases in a plasma and the damping of toroidal flow due to the stochastization is stronger than expected by the Rechester–Rosenbluth model.

## Results

### Experimental set-up

The large helical device (LHD) is a heliotron-type device for magnetic confinement of high-temperature plasmas. The LHD has three tangential neutral beams (NBs); two beams are used to change the direction of the plasma current from parallel (co-injection) to anti-parallel (counter-injection) with respect to the equivalent plasma current. The toroidal flow and ion temperature are measured with charge exchange spectroscopy^7^, while the rotational transform, *ı*/2*π*, and magnetic shear, *s*[=(*ρ*/*ι*)δ*ι*/δ*ρ*], at the rational surface (*ı*/2*π*=0.5) are measured with motional stark effect spectroscopy (MSE)^8^ in the LHD. There are three kinds of topology of the magnetic field in the plasma: the first is nested magnetic flux surfaces, the second is a stochastic magnetic field and the third is a magnetic island. The magnetic topology is identified by the characteristics of heat pulse propagation produced by modulated electron cyclotron heating (MECH), measured with electron cyclotron emission^9^. In the nested magnetic flux surfaces, the heat pulse propagates outwards on the time scale of the heat transport. In contrast, the heat pulse propagation becomes very fast due to the propagation along the magnetic field line in the stochastic region in the plasma.

### Observation of flow damping

**Figure 1:**
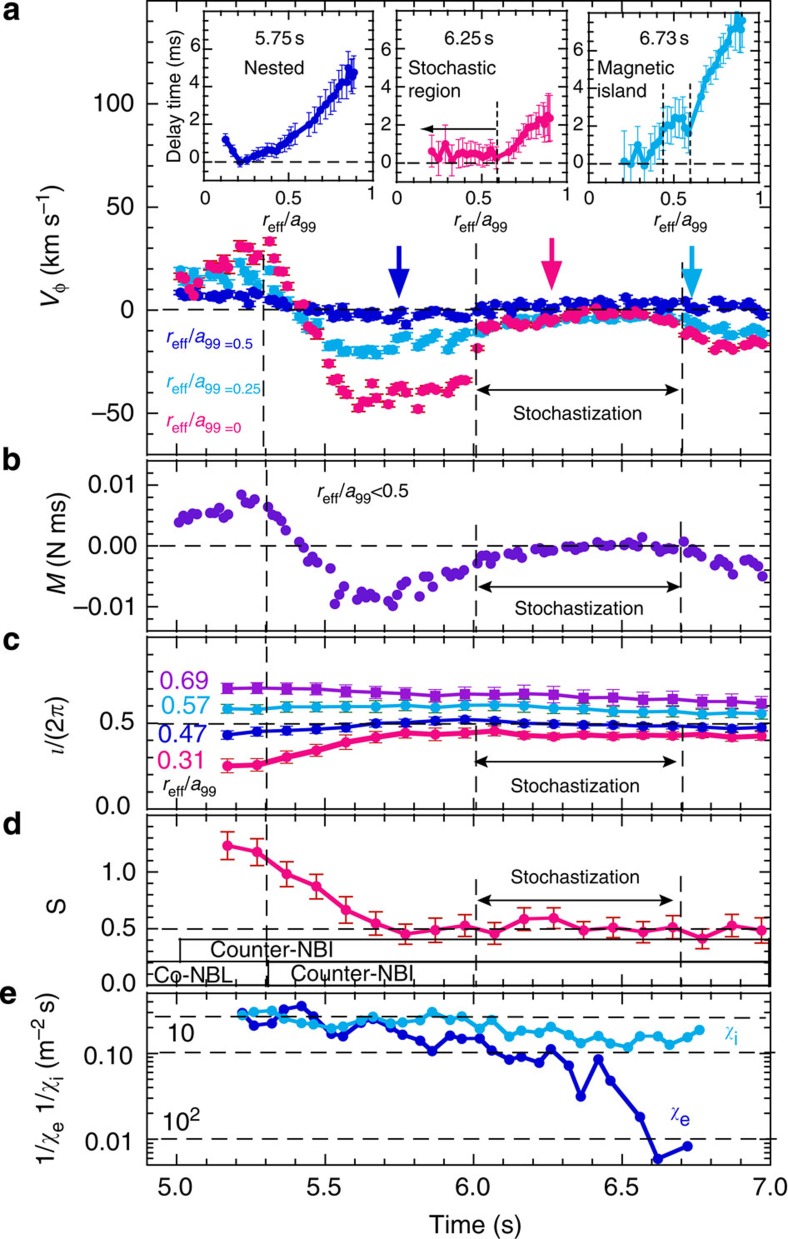
Time evolution of flow velocity and other plasma parameters. Time evolution of (**a**) toroidal flow velocity, *Vφ*, (**b**) angular momentum in the core (*r*_eff_/*a*_99_<1/2), (**c**) rotational transform, *ι*/2*π*, (**d**) magnetic shear, *s*, at the rational surface (*ι*/2*π*=0.5), and (**e**) inverse of the electron and ion thermal diffusivity, 1/*χ*_e_, 1/*χ*_i_, at *r*_eff_/*a*_99_=0.35 in the discharge where the direction of neutral beam injection (NBI) is switched from co- to counter-injection. Radial profiles of delay time of heat pulse produced by modulated electron cyclotron heating (MECH) at three time slices (*t*=5.45, 6.02, 6.72 s) are also plotted. The error bars of the delay times are standard deviations. The error bars of toroidal rotation are derived from the uncertainty of the fitting parameter of the charge exchange line emission to a Gaussian profile. The error bars of rotational transform and magnetic shear are derived from the standard deviations of the MSE signal.

Figure 1 shows the time evolution of toroidal flow, angular momentum, rotational transform, magnetic shear, and inverse of the electron and ion thermal diffusivity in the discharge, where the direction of the NB injection (NBI) is switched from co-injection to counter-injection (parallel to anti-parallel to the equivalent plasma current, which gives the poloidal field produced by the external coil current) at *t*=5.3 s. The vacuum magnetic axis is 3.6 m and the magnetic field strength is 2.75 T. The edge rotational transform decreases due to the NB current drive (NBCD) and the central rotational transform increases due to the inductive current; the magnetic shear at the *ι*/2*π*=0.5 rational surface starts to decrease and reaches the steady-state value of 0.5 at *t*=5.8 s after the switch of the NBI. This decrease of magnetic shear increases the magnetic island width and finally causes stochastization due to the overlapping of magnetic islands with higher modes^9^,^10^. The toroidal flow velocity changes its sign from positive (co-rotation) to negative (counter-rotation) and becomes steady state at a central toroidal flow velocity of −40 km s^−1^. An abrupt drop of the toroidal flow velocity is observed at *t*=6.0 s, although the NBI continues to be injected until *t*=7.3 s. The toroidal flow velocity starts to recover at *t*=6.7 s. The core angular momentum (*r*_eff_/*a*_99_<1/2) decreases at the stochastization, which suggests that this flow damping is not due to the increase in perpendicular viscosity but due to the direct loss of angular momentum.

The topology of the magnetic field is identified by the characteristics of the heat pulse propagation driven by MECH with a frequency of 25 Hz at the plasma center within *r*_eff_/*a*_99_<0.1. Here, *r*_eff_ is the averaged minor radius on a magnetic flux surface and *a*_99_ is the effective minor radius in which 99% of the plasma kinetic energy is confined, which is 0.63 m in this discharge. Just after the beam switch at *t*=5.45 s, the delay time of the heat pulse indicates that the magnetic topology is characterized by normal nested magnetic flux surfaces as seen in Fig. 1. At *t*=6.02 s, where abrupt drop of the toroidal flow velocity is observed, the delay time of the heat pulse propagation shows flattening in the plasma core (*r*_eff_/*a*_99_<0.57), which indicates the change of magnetic topology to a stochastic character (stochastization of the magnetic field). During the latter period in this discharge, at *t*=6.72 s, the radial profile of the delay time shows a small peak at *r*_eff_/*a*_99_~0.5, which indicates the magnetic island where the heat pulse propagates from the boundary to the O-point of the magnetic island, located at *r*_eff_/*a*_99_~0.5. There are two patterns of heat pulse propagation observed in the flat temperature region: one pattern is a very fast propagation as seen at *t*=6.02 s, and the other is simultaneous propagation at two separate points (*r*_eff_/*a*_99_~0.4 and 0.6) as seen at *t*=6.72 s. The former is clear evidence of the stochastization of the magnetic field, and the latter is consistent with a magnetic flux surface with a magnetic island. A negative slope of the time delay shows that the heat pulse propagates inward from the boundary of the magnetic island at *r*_eff_/*a*_99_~0.6 to the O-point, and a peaked delay profile indicates that the heat transport inside the magnetic island is comparable to or even better than that outside of the magnetic island^11^,^12^.

### Thermal diffusivity and viscosity

**Figure 2:**
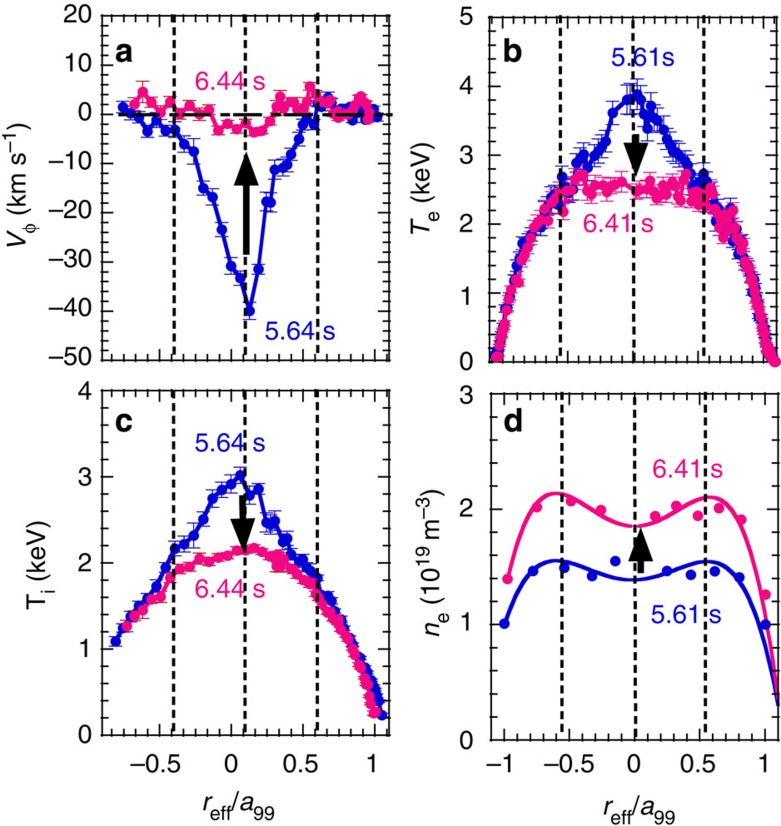
Radial profiles of flow velocity, temperatures, and density. Radial profiles of (**a**) toroidal flow velocity, (**b**) electron temperature, (**c**) ion temperature and (**d**) electron density before (*t*=5.64, 5.61 s) and after (*t*=6.44, 6.41 s) the stochastization of the magnetic field. The solid lines in the radial profiles of electron density are polynomial fit curves to data points. The error bars of toroidal rotation and ion temperature are derived from the uncertainty of the fitting parameter of the charge exchange line emission to a Gaussian profile. The error bars of electron temperature are derived from the standard deviations of the signal.

In the core plasma of the LHD, the electron thermal diffusivity evaluated from heat pulse is comparable to that evaluated from the power balance in the steady state^13^. These experimental results suggest that the heat pinch^14^ or other non-linearities of electron transport are small enough to be neglected in this experiment. Therefore, the effective transport coefficients (electron thermal diffusivity, ion thermal diffusivity and viscosity) are evaluated from the ratio of radial flux normalized by density to gradient for simplicity. Here, the radial flux of electron ion heat transport and momentum transport are calculated from the power deposition and torque profiles driven by the MECH and the NBIs. Figure 2 shows the radial profiles of toroidal flow velocity, electron temperature, ion temperature and electron density before (*t*=5.64 s, 5.61 s) and after (*t*=6.44 s, 6.41 s) the stochastization of the magnetic field. Before the stochastization, the toroidal flow velocity is very peaked at the plasma centre, because the toroidal viscosity due to helical ripple increases sharply towards the plasma edge and hence significant damping of the toroidal flow occurs there. After the stochastization, a clear flattening of the toroidal flow, ion temperature and electron temperature profiles is observed. Since the density profile is already flat even before the stochastization, the effect of stochastization on particle transport is not clear in this experiment. The increase of electron density is gradual and not due to the stochastization of the magnetic field.

Thermal diffusivity (defined as the ratio of the normalized heat flux to the temperature gradient) is evaluated for ion and electron transport, with a correction due to the slowing-down process^15^. The electron thermal diffusivity (*χ*_e_) at *r*_eff_/*a*_99_=0.35 (*r*_eff_=0.2 m) increases by more than one order of magnitude (*χ*_e_=4.1±1.2 m^2^ s^−1^→>10^2^ m^2^ s^−1^), while the ion thermal diffusivity (*χ*_i_) increases only by a factor of 1.8 (*χ*_i_=3.8±0.3 m^2^ s^−1^→6.9±0.9 m^2^ s^−1^). The electron and ion thermal diffusivity in the stochastic region can be evaluated as 
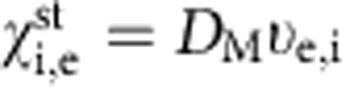
 using the Rechester–Rosenbluth model^6^. Here *υ*_e_ and *υ*_i_ are the thermal velocities of electron and ion, respectively, and *D*_M_ is the diffusion of the field line, defined by 
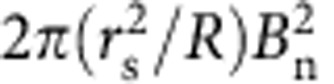
, *r*_s_ is the radius of the resonant surface and *B*_n_ (=(*RB*_r_)/(*r*_s_*B*_*φ*_)) is the normalized perturbation field^16^. Then *χ*_e_ in the stochastic region is expected to be much larger than that of the ions by (*m*_i_/*m*_e_)^1/2^ because of its larger thermal velocity^5^,^17^. The thermal diffusivity in the stochastic region χ^st^ evaluated from similar discharges^9^ is 2.5±0.5 × 10^2^ m^2^ s^−1^ for electrons and 6±1 m^2^ s^−1^ for ions, which is consistent with this experimental observation. The magnitude of the electron thermal diffusivity is comparable to that estimated from the power balance and also predicted by the analytic formula of the Rechester–Rosenbluth model^6^ in RFX^1^ and in MST^3^ experiments (10^2^–10^3^ m^2^ s^−1^).

The viscosity coefficient of the toroidal flow (*μ*_*φ*_) is similar to *χ*_*i*_ before the stochastization, which indicates that both momentum and ion heat transport are dominated by turbulence transport. However, the increase in *μ*_*φ*_ is by a factor of 5 (*μ*_*φ*_=4.0±1.6 m^2^ s^−1^→21±5 m^2^ s^−1^), which is much larger than that in ion thermal transport. This fact suggests that the damping of the toroidal flow is not only due to the increase of the viscosity coefficient. Because the toroidal flow velocity is much more peaked in the core region (*r*_eff_/*a*_99_<0.5), where the stochastization takes place, than the ion and electron temperature, the change in toroidal flow is most significant at the topology bifurcation from nested flux surface to the stochastic magnetic field.

### Physics mechanism of flow damping

**Figure 3:**
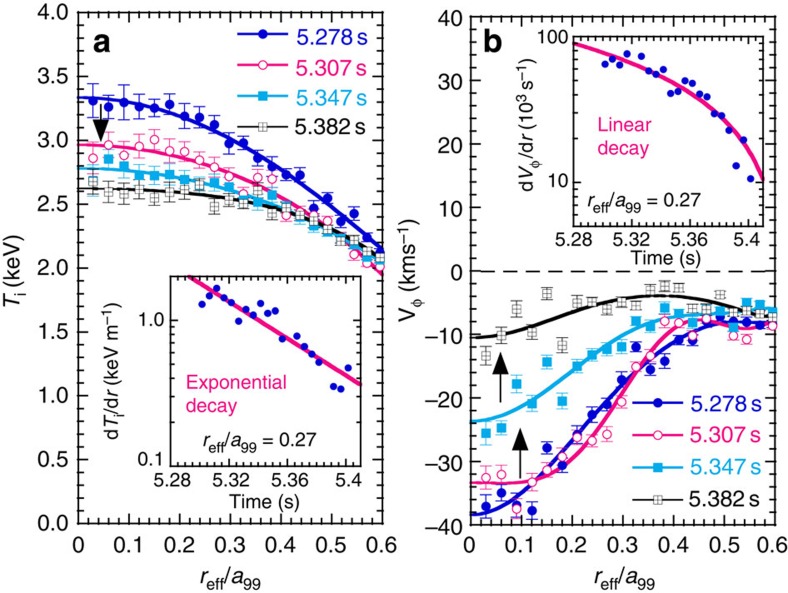
Decay of ion temperature and flow velocity. Radial profiles of (**a**) ion temperature and (**b**) toroidal flow velocity in the core region (*r*_eff_/*a*_99_<0.6) during the stochastization of the magnetic field. The decay of the ion temperature gradient and the toroidal flow velocity shear at *r*_eff_/*a*_99_=0.27 are also plotted in log-scale. The solid lines in the radial profiles of ion temperature and toroidal rotation are polynomial fit curves to data points and the solid lines in the time evolution of the ion temperature gradient and velocity shear in the log-plot are the exponential curve and linear curve to fit data points. The error bars of toroidal rotation and ion temperature are derived from the uncertainty of the fitting parameter of the charge exchange line emission to a Gaussian profile.

In this section, the physics mechanism of flow damping is discussed. The large effective Prandtl number observed during stochastization (*μ*_*φ*_/*χ*=3) discussed in the previous section suggests the existence of an additional damping mechanism of the toroidal flow due to stochastization of the magnetic field. Figure 3 shows radial profiles of the ion temperature and toroidal flow velocity in the core region of *r*_eff_/*a*_99_<0.6, and the decay of the ion temperature gradient and the toroidal flow velocity shear during the decay phase at *r*_eff_/*a*_99_=0.27. The ion temperature profiles show a prompt flattening after the stochastization (the drops in ion temperature occur earlier and faster over time); however, the toroidal flow velocity profiles show a damping after the drop of ion temperature in the time scale of 100 ms. The decay rate of the central ion temperature becomes smaller with time (δ*T*_i_/δ*t*(5.278–5.307 s)>δ*T*_i_/δ*t*(5.307–5.347 s)~δ*T*_i_/δ*t*(5.347–5.382 s)), while that of the toroidal rotation becomes larger and then constant with time (δ*V*_*φ*_/δ*t*(5.278–5.307 s)<δ*V*_*φ*_/δ*t*(5.307–5.347 s)~δ*V*_*φ*_/δ*t*(5.347–5.382 s)). There is a clear difference in the decay of the ion temperature gradient and the toroidal flow velocity shear observed. The toroidal flow shear shows a linear decay, while the ion temperature gradient shows an exponential decay, which is indicated by the solid lines in Fig. 3a,b in log-scale. The toroidal flow shear decreases more than the linear curve at *t*~5.4 s, as seen in Fig. 3b. This experimental observation (linear decay) cannot be explained by the increase in the diffusive term of the momentum transport, which should be proportional to the velocity shear, and it suggests a new damping mechanism.

### Radial propagation of stochastization

**Figure 4:**
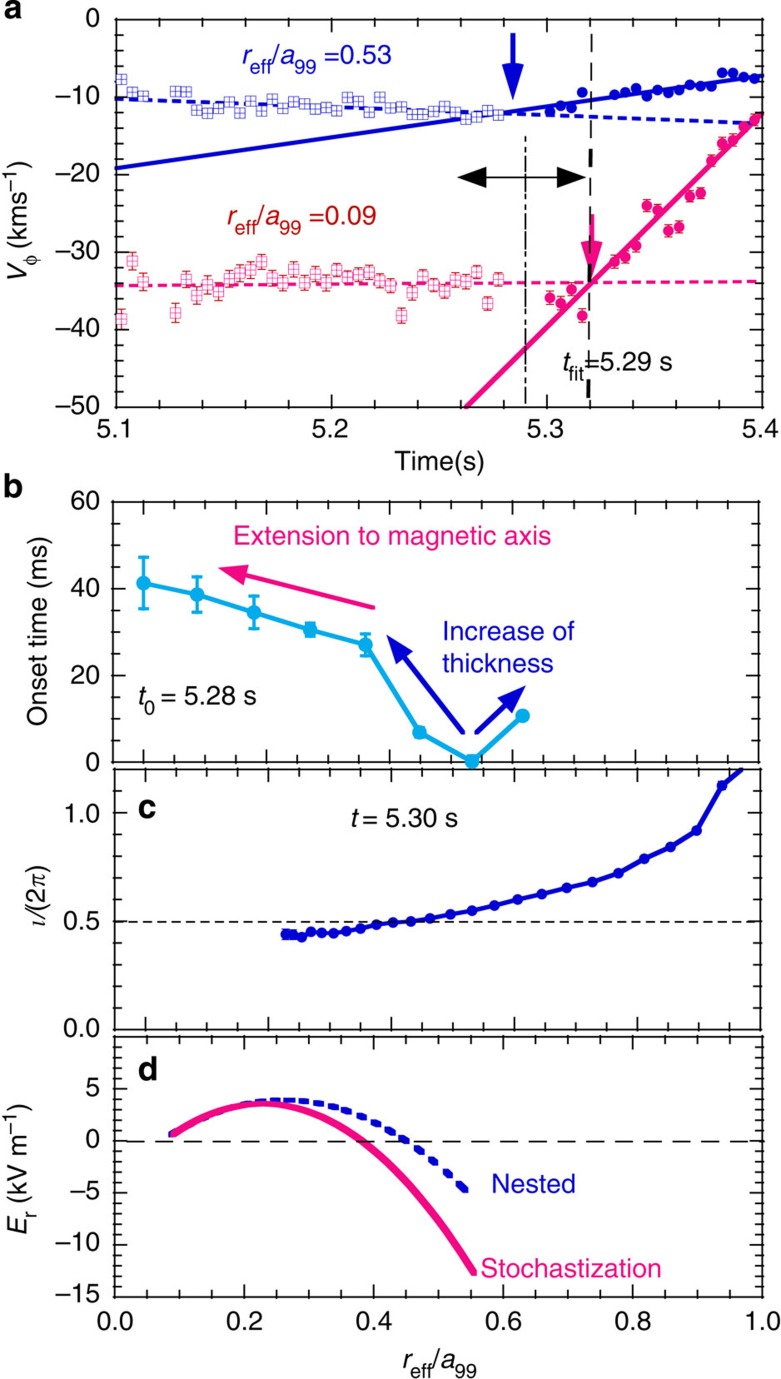
Radial propagation of stochastization. (**a**) Time evolution of toroidal flow velocity and two liner fitting lines in the case of *t*_fit_=5.29 s at *r*_eff_/*a*_99_=0.09 and 0.53 during the stochastization of the magnetic and radial profiles of (**b**) onset time of flow damping associated with the stochastization of magnetic field, (**c**) rotational transform *ι*/2*π*, and (**d**) radial electric field before stochastization (with nested magnetic flux surface) and after the stochastization measured with HIBP in a similar discharge. The onset time (intersections of two lines) is indicated with the arrows in (**a**). The typical error bar of the measurements of rotational transform in (**c**) is 0.01. Because the time of the stochastization itself is unknown before the fitting and the intersection depends on the separation of the data (namely *t*_fit_), *t*_fit_ is scanned from well before (*t*=5.26 s) to well after (*t*=5.32 s) the stochastization by 60 ms. The error bars are determined from the standard deviation in this scan.

It is an interesting issue how the stochastic region develops in time during the stochastization. In order to study the radial propagation of stochastization, the radial profile of the time taken for the abrupt drop of toroidal flow velocity is studied. Figure 4 shows the time evolution of the toroidal flow near the *ι*/2*π*=0.5 surface and magnetic axis, and a radial propagation of the onset of the flow damping and the radial profile of the rotational transform measured with the MSE. As discussed earlier, the decay of toroidal flow velocity can be fitted well with a linear line. Therefore we can derive the onset time of flow damping from the intersection of two lines of linear fitting to the data before (*t*<*t*_fit_) and after (*t*>*t*_fit_) the stochastization. Because the time of the stochastization itself is unknown before the fitting and the intersection depends on how the data are separated (namely *t*_fit_), *t*_fit_ is scanned from well before (*t*=5.26 s) to well after (*t*=5.32 s) the stochastization by 60 ms, and the onset time and its error bars are determined from the average value and standard deviation in this scan. Please note that the onset time is insensitive to *t*_fit_. These results show that the stochastization starts near the rational surface of *ι*/2*π*=0.5 at *r*_eff_/*a*_99_=0.45–0.55 and propagates radially in two time scales. The thickness of the stochastic region increases slowly up to a quarter of the plasma minor radius (*r*_eff_/*a*_99_=0.36–0.62), and then a rapid extension of the stochastic region to the magnetic axis takes place. The sudden extension of the stochastic region to the magnetic axis observed in this experiment indicates the non-linear growth of the perturbation field causing stochastization, which was proposed in the major disruption or sawtooth crash model^16^,^18^.

## Discussion

The change in a radial electric field associated with stochastization is plotted in Fig. 4d. The positive radial electric field in the core region (*r*_eff_/*a*_99_<0.4) decreases and the negative radial electric field outside this region increases after stochastization of the magnetic field. The change in the radial electric field is more significant near the rational surface, where the dominant modes are resonant, while the flattening of the electron temperature is observed in the whole core region. This observation is consistent with the fact that the transport enhanced due to the stochastization of magnetic field is ambipolar except for the region near locations where the dominant modes are resonant^19^. Although the neoclassical transport is sensitive to the radial electric field, the effect of the radial electric field on transport is relatively small because the electron heat transport is dominated by turbulence transport in this plasma.

In this experiment, the abrupt damping of the toroidal flow due to stochastization of the magnetic field is observed when the magnetic shear drops to 0.5 after the switch of the NBI from co-injection to counter-injection. The stochastization starts near the rational surface of *ι*/2*π*=0.5 and the stochastic region develops to the magnetic axis in two time scales: one is a slow increase of the stochastization width and the other is a fast extension to the magnetic axis. After the stochastization of the magnetic field, the increase of *χ*_e_ is much larger than that of the ions (*χ*_e_/*χ*_i_>15) because of the difference in thermal velocity, which is consistent with the Rechester–Rosenbluth model (~40) (ref. 6). On the other hand, the flow damping observed cannot be explained by this model and there are clear differences in the decay between ion temperature and toroidal flow velocity, which suggests that the damping of flow is due to the change in the non-diffusive term of momentum transport associated with the stochastization of the magnetic field.

One of the candidates for a new flow damping mechanism is the change in the non-diffusive term of toroidal momentum transport^20^ due to stochastization of the magnetic field. The other candidate is a toroidal momentum pinch as a direct electromagnetic effect, which is also proportional to the ion temperature gradient^21^. Here a stochastization of the magnetic field may reduce the phase correlation between magnetic vector potential and electrostatic potential, thus resulting in reduction of the momentum pinch. These electromagnetic effects on toroidal momentum transport (momentum pinch) become strong in the plasma with large ion temperature gradient and decrease the effective Prandtl number significantly (even to negative values in the case of small density gradients). In our experiment, the Prandtl number before the stochastization is close to unity and the momentum pinch effect is expected to be small. After the stochastization, the ion temperature gradient, which causes the momentum pinch, becomes even smaller. This flow damping mechanism is a strong candidate for the angular momentum loss due to the magnetic island disruption in tokamaks, and should also be important in the solar flare, where the magnetic stochastization (overlapping of magnetic islands) is one of the candidates to explain the fast time scale of magnetic field reconnection^22^,^23^.

## Methods

### Large helical device

LHD is a heliotron-type device for magnetic confinement of high-temperature plasmas within a magnetic field, *B*, of 2.7 T at the magnetic axis in the vacuum field, with a major radius, *R*_ax_, and effective minor radius, *r*_eff_, of 3.6 and 0.63 m, respectively. In this experiment, the plasma density is 1–2 × 10^19^ m^−3^ and the central temperature is in the range of 2–4 keV. The LHD is equipped with three tangential NBs in the opposite injection direction (two counterclockwise and one clockwise) and electron cyclotron heating (ECH). The NBs are applied for both electron and ion heating, while the ECH is applied for electron heating focused at the magnetic axis in the plasma. The NBs are also used to control the magnetic shear with the toroidal current driven by the NB.

### NB current drive

NB current drive (NBCD) is one of the useful tools to drive toroidal current. The total plasma current driven by the NB is in the range of ~100 kA (counter-direction) to 50 kA (co-direction), which is only 3–6% of the equivalent plasma current (1.8 MA) produced by the external helical coils. However, the time scale in the change of total current is longer than the beam pulses, and the inductive current in the direction opposite to the toroidal current due to NBCD in the core region plays an important role in this experiment. Therefore, by switching the direction of the NBCD during the discharge, the magnetic shear near the plasma core can be controlled. In this experiment, the direction of the injected NB switches from parallel (co-direction) to antiparallel (counter-direction) with respect to the equivalent plasma current, and magnetic shear decreases effectively (from 1.3 to 0.5) after the injection of the NB in the counter-direction.

### Modulated ECH

The heat pulse propagation experiment is a useful tool for identifying the magnetic topology in toroidal plasmas. In a magnetic flux surface with a magnetic island, the heat pulse shows bi-directional slow propagation. This is because the perturbation is felt simultaneously at two points separated in radius, which can be interpreted as a surface equilibration. On the other hand, the heat pulse shows very fast propagation in the magnetic flux surface with stochastization due to the heat pulse propagation along the magnetic field in the time scale of thermal velocity. Recently MECH has been applied to investigate the characteristics of heat pulse propagation. In this experiment, MECH with a frequency of 25 Hz focused at the plasma centre is applied.

### Thermal diffusivity and viscosity

Thermal diffusivity and viscosity are evaluated from the ratio of the heat flux and momentum flux to the temperature gradient and velocity gradient. The heat flux and momentum flux are calculated from the heating and torque profiles from NBs and ECH using the FIT-3D code, where the steady-state solution of the Fokker–Planck equation is solved based on the birth profile of fast ions calculated by the Monte–Carlo method with the radial redistribution of fast ions due to prompt orbit effects with a correction due to the slowing-down process.

## Author contributions

K.I. proposed and performed the experiments, analysed the data and wrote the manuscript. M. Yoshinuma. and H.T. provided toroidal flow data and heat pulse propagation data, respectively. T.K. analysed the heat pulse propagation data driven by MECH. C.S. and M. Yokoyama developed analysis tools to derive the thermal diffusivity and viscosity. A.S. provided radial electric field data. K.N. supported the NBCD in this experiment. S.I. provided the analysis code of the heat pulse propagation. K.I. provided the theoretical model.

## Additional information

**How to cite this article:** Ida, K. *et al.* Flow damping due to stochastization of the magnetic field. *Nat. Commun.* 6:5816 doi: 10.1038/ncomms6816 (2015).

## References

[b1] Bartiromo R. *et al.* Core transport improvement during poloidal current drive in the RFX reversed field pinch. Phys. Rev. Lett. 82, 1462–1465 (1999).

[b2] Innocente P. *et al.* Transport and confinement studies in the RFX-mod reversed-field pinch experiment. Nucl. Fusion 47, 1092–1100 (2007).

[b3] Biewer T. M. *et al.* Electron heat transport measured in a stochastic magnetic field. Phys. Rev. Lett. 91, 045004 (2003). 12906670 10.1103/PhysRevLett.91.045004

[b4] Sarff J. S. *et al.* Tokamak-like confinement at a high beta and low toroidal field in the MST reversed field pinch. Nucl. Fusion 43, 1684–1692 (2003).

[b5] Fiksel G. *et al.* Measurement of magnetic fluctuation-induced heat transport in tokamaks and RFP. Plasma Phys. Control. Fusion 38, A213–A225 (1996).

[b6] Rechester A. B. & Rosenbluth M. N. Electron heat transport in a tokamak with destroyes magnetic surfaces. Phys. Rev. Lett. 40, 38–41 (1978).

[b7] Yoshinuma M. *et al.* Charge-exchange spectroscopy with pitch-controlled double-slit fiber bundle on LHD. Fusion Sci. Technol. 58, 375–382 (2010).

[b8] Ida K. *et al.* Measurements of rotational transform with motional stark effect spectroscopy. Fusion Sci. Technol. 58, 383–393 (2010).

[b9] Ida K. *et al.* Topology bifurcation of a magnetic flux surface in magnetized plasmas. New J. Phys. 15, 013061 (2013).

[b10] Ida K. *et al.* Bifurcation phenomena of a magnetic island at a rational surface in a magnetic-shear control experiment. Phys. Rev. Lett. 100, 045003 (2008). 18352289 10.1103/PhysRevLett.100.045003

[b11] Inagaki S. *et al.* Observation of reduced heat transport inside the magnetic island O point in the large helical device. Phys. Rev. Lett. 92, 055002 (2004). 14995316 10.1103/PhysRevLett.92.055002

[b12] Yakovlev M. *et al.* Heat pulse propagation across the rational surface in a large helical device plasma with counter-neutral beam injection. Phys. Plasmas 12, 092506 (2005).

[b13] Inagaki S. *et al.* Comparison of transient electron heat transport in LHD helical and JT-60U tokamak plasmas. Nucl. Fusion 46, 133–141 (2006).

[b14] Luce T. *et al.* Inward energy transport in tokamak plasmas. Phys. Rev. Lett. 68, 52–55 (1992). 10045110 10.1103/PhysRevLett.68.52

[b15] Lee H. *et al.* Dynamic transport study of heat and momentum transport in a plasma with improved ion confinement in the large helical device. Plasma Phys. Control. Fusion 55, 014011 (2013).

[b16] Lichtenberg A. J. *et al.* The role of stochasticity in sawtooth oscillations. Nucl. Fusion 32, 495–512 (1992).

[b17] Itoh S.-I. *et al.* A model of giant ELMs. Plasma Phys. Control. Fusion 38, 527–549 (1996).

[b18] Itoh K. *et al.* Model of the major disruption in tokamaks. Nucl. Fusion 32, 1851–1855 (1992).

[b19] Terry P. W. *et al.* Ambipolar magnetic fluctuation-induced heat transport in toroidal devices. Phys. Plasmas 3, 1999–2005 (1996).

[b20] Ida K. *et al.* Spontaneous toroidal rotation driven by the off-diagonal term of momentum and heat transport in the plasma with the ion internal transport barrier in LHD. Nucl. Fusion 50, 064007 (2010).

[b21] Mahmood A., Eriksson A. & Weiland J. Electromagnetic effects on toroidal momentum transport. Phys. Plasmas 17, 122310 (2010).

[b22] Parker E. N. The solar-flare phenomenon and the theory of reconnection and annihilation of magnetic fields. ApJS 8, 177–211 (1963).

[b23] Yokoyama T. & Shibata K. What is the condition for fast magnetic reconnection? ApJ 436, L197–L200 (1994).

